# Age influences susceptibility of brain capillary endothelial cells to La Crosse virus infection and cell death

**DOI:** 10.1186/s12974-021-02173-4

**Published:** 2021-06-03

**Authors:** Rahul Basu, Vinod Nair, Clayton W. Winkler, Tyson A. Woods, Iain D. C. Fraser, Karin E. Peterson

**Affiliations:** 1 Research and Technologies Branch, Rocky Mountain Laboratories, NIAID, NIH, 903 S. 4th Street, MT 59840 Hamilton, USA; 2 Signaling Systems Section, Laboratory of Immune System Biology, National Institute of Allergy and Infectious Diseases, National Institutes of Health, 4 Memorial Drive, Bethesda, MD 20892 USA

**Keywords:** Brain capillary endothelial cells, Blood-brain barrier, Cytopathic effect, La Crosse virus, Vascular leakage

## Abstract

**Background:**

A key factor in the development of viral encephalitis is a virus crossing the blood-brain barrier (BBB). We have previously shown that age-related susceptibility of mice to the La Crosse virus (LACV), the leading cause of pediatric arbovirus encephalitis in the USA, was associated with the ability of the virus to cross the BBB. LACV infection in weanling mice (aged around 3 weeks) results in vascular leakage in the olfactory bulb/tract (OB/OT) region of the brain, which is not observed in adult mice aged > 6–8 weeks. Thus, we studied age-specific differences in the response of brain capillary endothelial cells (BCECs) to LACV infection.

**Methods:**

To examine mechanisms of LACV-induced BBB breakdown and infection of the CNS, we analyzed BCECs directly isolated from weanling and adult mice as well as established a model where these cells were infected in vitro and cultured for a short period to determine susceptibility to virus infection and cell death. Additionally, we utilized correlative light electron microscopy (CLEM) to examine whether changes in cell morphology and function were also observed in BCECs in vivo.

**Results:**

BCECs from weanling, but not adult mice, had detectable infection after several days in culture when taken ex vivo from infected mice suggesting that these cells could be infected in vitro. Further analysis of BCECs from uninfected mice, infected in vitro, showed that weanling BCECs were more susceptible to virus infection than adult BCECs, with higher levels of infected cells, released virus as well as cytopathic effects (CPE) and cell death. Although direct LACV infection is not detected in the weanling BCECs, CLEM analysis of brain tissue from weanling mice indicated that LACV infection induced significant cerebrovascular damage which allowed virus-sized particles to enter the brain parenchyma.

**Conclusions:**

These findings indicate that BCECs isolated from adult and weanling mice have differential viral load, infectivity, and susceptibility to LACV. These age-related differences in susceptibility may strongly influence LACV-induced BBB leakage and neurovascular damage allowing virus invasion of the CNS and the development of neurological disease.

**Supplementary Information:**

The online version contains supplementary material available at 10.1186/s12974-021-02173-4.

## Background

The La Crosse virus (LACV) is a tri-segmented, negative-sense RNA arbovirus, belonging to the family Bunyaviridae [1]. In most people, LACV infection causes very mild, febrile syndromes without significant neurological disease [2]. However, in some cases, LACV can infect the central nervous system (CNS) and induce neurological disease, ranging from behavioral changes, cognitive defects, and seizures to coma or death. Interestingly, the neurological disease caused by LACV mostly occurs in children under the age of 16, indicating an age-related susceptibility in children [3]. A similar age-dependent LACV-induced neurological disease is observed in the C57Bl/6 mouse model. In this model, weanling mice (aged ~ 3 weeks) are susceptible to peripheral (intraperitoneal; i.p.) inoculation of LACV while adult mice (aged ≥ 6–8 weeks) are resistant [2]. The neurological disease that develops following i.p. inoculation of weanling mice is associated with high viral titers in the CNS. Adult mice can develop neurological disease when the virus is directly delivered into CNS by intracranial (i.c.) injection, which indicates that the age-related resistance to LACV is conferred by either controlling viral replication in the periphery, or an inability of the virus to gain access to the CNS [2, 4]. Studies have shown differences in innate and adaptive immune responses between weanling and adult mice that contribute to their differences in susceptibility to viral encephalitis [5]. However, whether age-related differences influence the permeability of the blood-brain barrier (BBB) or the function of brain capillary endothelial cells (BCECs) remains unknown.

The importance of the BBB in limiting virus infections of the CNS was first shown with vesicular stomatitis virus in the late 1930s [6]. This early study showed brains of adult animals had a sort of “impermeable barrier” that stopped viral infection and encephalitis in adult animals, which was not observed in young animals. Since that time, additional studies have demonstrated a role of the BBB in protecting the CNS from toxins and infectious agents including arboviruses [7–9]. As a protective barrier, the BBB is comprised of BCECs, astrocytes, basement membrane, and pericytes. Together these cells, along with surrounding neurons and microglia form the neurovascular unit (NVU). The cell junction proteins present in these cells, namely tight junction (TJ), gap junction (GJ), and adherens junction (AJ) proteins, have a critical role in forming the BBB to protect the CNS from invading pathogens and toxic substances [10, 11]. Previous studies from our laboratory have shown that the breakdown of the BBB is a key mechanism by which LACV gains access to the CNS in young mice. Specifically, brain capillary endothelial cells (BCECs) located in the olfactory bulb (OB) and anterior olfactory tract (AOT) regions were found to allow entry of LACV at approximately 3 days post infection (dpi) [4]. Thus, the response of BCECs to LACV may be a key regulator in allowing virus access to the brain. Understanding how LACV affects BCECs, and whether there are age-related differences that influence BBB breakdown, is a pivotal factor in developing strategies to prevent viral encephalitis.

Viral infection and entry procedures can involve morphological and functional changes in the BCECs. These procedures include alteration in the BBB proteins or their regulators like TJ proteins and matrix metalloproteinases [12, 13]. Several viruses alter GJ or TJ proteins during infection [14, 15]. Viruses like the hepatitis C virus and human T lymphotropic virus also utilize TJ proteins (claudins and occludin) for viral entry [16, 17]. Several viruses also infect ECs or harbor viral RNA and can induce cell death. For example, HIV and measles virus (MV) are reported to promote apoptosis in brain endothelial cells [18, 19]. As LACV entry involves active leakage through the BCECs, specifically in the weanling mice, the age-related differences in the BCECs have pivotal importance and warrant further study in relation to LACV entry into the CNS. Along with that, several cytoskeletal proteins and their binding partners in the BCECs are differentially regulated during LACV infection. These proteins mainly involve actin cytoskeletal remodeling and are predicted to be involved in the alteration of cell junction proteins [4]. For example, actin can directly bind to ZO1 and is able to control the barrier-forming properties of BCECs [20]. Hence, it is crucial to understand the infection-induced or age-associated intrinsic differences among BCECs and their functional barrier properties, which might be an underlying mechanism of LACV-induced encephalitis. One study showed age-related differences in encephalitis during Chikungunya virus infections [21] but the age-dependent pathological changes and susceptibility remain elusive. Thus, in this study, we established a model to portray the differences between weanling and adult BCECs in LACV infection and investigated the putative mechanism by which LACV selectively induces leakage through the weanling BCECs.

The mechanism by which LACV gains access to the CNS is not well understood. One report indicated that LACV was detected by immunohistochemistry (IHC) within the brain capillaries present in brain biopsy samples [22], although other studies in mouse models have not observed BCEC infection in vivo [4]. A case report observed vascular injury resulting from virus-induced encephalitis during acute illness [23]. However, whether the BCECs were directly infected was not clear from these clinical studies. In our previous studies using LACV-infected young mice, we did not detect virus infection of BCECs by either IHC of brain tissue or confocal microscopy of direct ex vivo-isolated BCECs, and we did not detect virus proteins in BCEC homogenates [4]. However, focal areas of vascular leakage were observed in brain capillaries in the OB and AOT region, but not the cortex of those same mice. No vascular leakage was found in any region of the brain in LACV-infected adult mice, suggesting that the BBB may be more susceptible to virus-induced damage in young animals.

In the current study, we examined if there were age-related differences in the BCECs that affect LACV pathogenesis in those cells. We used ex vivo-isolated as well as in vitro-cultured BCECs from weanling and adult mice to examine their response to LACV. We found age-related differences in susceptibility of BCECs to LACV, as well as morphological changes and cell death. These results suggest that there are intrinsic age-related properties of BCECs that are maintained after isolation and in vitro culture and that these properties determine their response to LACV infection.

## Methods

### Infection of mice with LACV

All animal studies were conducted under animal protocol RML-2018-018-E adhering to the Principles of Laboratory Animal Care and in accordance and approval by the NIH/NIAID/RML Institutional Animal Care and Use Committee. C57BL/6 mice obtained from Jackson Laboratories were maintained in a breeding colony at RML. LACV human 1978 stock, a kind gift from Dr. Richard Bennett, was used to study LACV pathogenesis in the mice [1]. Weanling (aged ~ 3 weeks) and adult (aged > 6–8 weeks) were inoculated intraperitoneally (i.p.) with 2000 PFU of LACV prepared in 200 μl phosphate-buffered saline (PBS). Mock-inoculated mice were maintained in parallel, which are represented as 0 dpi. The brains were isolated from LACV and mock-inoculated mice at 1, 2, and 3 days post infection (dpi).

### Isolation of microvessel fragments

Primary BCECs from microvessel fragments were isolated from weanling and adult mice and cultured as previously described [24, 25]. Briefly, mice were transcardially perfused with PBS to remove circulating blood. Brain tissue was removed, cut into small pieces, then digested with 10 mg/ml of collagenase (Worthington Biochemical) in Dulbecco’s modified Eagle’s medium (DMEM; Sigma) in a shaker for 1 h at 37 °C. The digested tissues were separated from white matter lipids and other cellular debris by centrifugation (at 1000*g* for 20 min) in the presence of 20% bovine serum albumin solution prepared in DMEM. The resultant pellets were further digested in the presence of 10 mg/ml of collagenase/dispase (Roche Applied Science) in DMEM for 45 min to 1 h at 37 °C. The microvessel fragments obtained from the enzyme-digested pellets were separated on a 33% continuous Percoll gradient (1000 g, 10 min), collected, and washed twice in DMEM before using them for ex vivo and in vitro experiments. For analysis and comparison of the olfactory bulb (OB) and cortex from the brain, those specific regions were isolated using fine-tip forceps. A similar protocol was followed to isolate BCECs from other brain regions. Primary endothelial cultures were positive for endothelial cell markers like ZO1 or claudin 5 (visualized by immunofluorescence assay), with no detection of negative cells, consistent with findings from others with this established protocol [25, 26].

### Selection and culture of BCECs

The microvessel fragments were plated on 24-well plates or chambered glass slides coated with collagen type IV (Sigma) and human fibronectin (Sigma) for RNA isolation or immunofluorescence assay (IFA). Cultures were maintained in DMEM supplemented with 20% fetal bovine serum, 1% penicillin/streptomycin, 1% glutamine, 1 ng/ml of fibroblast growth factor-2 (R&D Systems), and 4 μg/ml of puromycin (Sigma) for BCEC selection in a humidified 5% CO_2_, 95% air atmosphere at 37 °C. Four to 6 days following plating, BCEC cultures visually contained continuous monolayers of spindle-shaped cells consistent with previously published findings [25]. BCEC cultures showed strong presence of endothelial cell markers such as claudin 5 or zona occludens 1 (ZO1). Primary cells were not subcultured for either ex vivo or in vitro experiments to maintain cells as close to in vivo conditions as possible. For ex vivo experiments, BCECs were isolated from LACV-infected weanling or adult mice and then maintained in BCEC culture medium until confluence with no medium changes. For in vitro experiments, confluent monolayers of BCECs isolated from uninfected weanling or adult mice were inoculated with different multiplicities of infection (MOIs) of LACV. After 1 h, the viral inoculum was removed and cells were washed once before addition of new media. Cells were then analyzed at different time points post infection. Mock-inoculated sets were maintained in parallel with similar treatment except addition of mock supernatant.

### RNA extraction and real-time PCR

Total RNA was isolated from whole brain tissue, ex vivo-isolated BCECs or cultured BCECs using RNA isolation kits from Zymo Research, using the manufacturer’s protocol. Total RNA was treated with DNase I (Invitrogen) for 30 min at 37 °C and was purified over RNA cleanup columns (Zymo Research). cDNAs were prepared from cleaned up RNA samples using the iScript reverse transcription kit (Bio-Rad Laboratories) following the manufacturer’s instructions, followed by fivefold dilution of cDNA samples. These samples were used for gene expression analysis by quantitative real-time PCR using SYBR Green SuperMix with ROX (Bio-Rad Laboratories). For all the analyses, GAPDH was used as an internal control.

### Plaque assay

The supernatants from the infected as well as mock-inoculated BCEC cultures were collected at 24, 48, and 72 hours post infection (hpi) for inoculations of 0.1 and 10 MOI and at 18, 36, and 48 hpi for inoculations of MOI 1. Plaque assays were performed using serial dilutions of the cell culture supernatant using monolayers of Vero cells as described previously [27]. Briefly, confluent Vero cells were incubated with different dilutions of supernatants in DMEM containing 2% FBS 1% penicillin/streptomycin. After 1 h, MEM containing 1.5% carboxymethyl cellulose was overlaid onto the cultures which were then incubated at 37 °C in a humidified 5% CO2, 95% air atmosphere for 5 days. Cultures were then fixed using 10% formalin and washed with water. The LACV plaques were visualized using 0.35% crystal violet solution and counted. All statistical analyses using GraphPad Prism 8 software.

### Immunofluorescence assay

Immunofluorescence studies were done according to a protocol described previously [28]. For standard immunofluorescence, primary BCECs were plated on a multi-well chambered glass slide. The cells were fixed using 4% paraformaldehyde (PFA), followed by permeabilization of the cells with PBS containing 0.5% Triton X-100. The cells were further blocked with PBS containing 0.5% Triton X-100 and 5% heat-inactivated donkey serum and then incubated with primary antisera diluted in blocking solution for 1 h. To remove nonspecifically bound antibodies, cells were washed and then labeled with secondary antisera diluted in blocking solution for 1 to 1.5 h. Finally, the cells were washed with PBS and incubated with DAPI stain for 10 min. After staining the nuclei, the cells were washed again, and a glass coverslip was mounted on top using Prolong Gold antifade reagent (Thermo Scientific). The images were acquired using a Nikon Ti2 microscope or a Zeiss confocal microscope (LSM710) and processed with ImageJ or Nikon Ti2 NIS elements software. For each experimental set, the background fluorescence intensity of viral staining was removed using the mock-inoculated samples and upon thresholding, the sum of the total intensity was divided by total nucleus area to obtain the total intensity of target protein per nucleus. Each point, in the quantification graphs, represents a machine-defined, randomized imaging field. The images were acquired at × 10 or × 40 objective magnification.

### Correlative light-electron microscopy

Mock-inoculated or LACV-infected mice (3 dpi) were intravenously (IV) injected with 100 nm fluorosphere beads (Thermo Scientific) via the retro-orbital route. After 30 min, mice were transcardially perfused with PBS, followed by a 3% PFA/1% glutaraldehyde mixture. Brains were then isolated and drop-fixed in the 3% PFA/1% glutaraldehyde mixture for an additional 24 to 48 h. After a couple of rinses in buffer, the OBs were dissected out in PBS and 50-μm serial sections were taken using a vibratome (Leica). These sections were screened for the presence of fluorosphere beads using a Zeiss LSM710 confocal microscope to detect regions of vascular leakage. Sections showing the presence of fluorescent beads were prepared for transmission electron microscopy (TEM) using standard protocols and imaged on an 80-kV Hitachi H7800 microscope (Hitachi High-Technologies America Inc.) using a Hamamatsu Orca HR camera (Advanced Microscopy Techniques, Corp.). Images acquired are from a single experiment.

### Cell viability assay

BCECs were plated and infected as described above. After removing the viral inoculum, Cytotox Green reagent was diluted in BCEC specific medium according to the guidelines of the manufacturer (Essen Bioscience) to a final concentration of 250 nM. The cells were imaged every 3 h for green fluorescence (marker of cell death) and phase contrast with an IncuCyte (Essen Bioscience) by taking 9 images per well until 72 hpi, using the × 10 objective. The measured confluence and fluorescent intensity using IncuCyte S3 software and live cell images were converted to a representative video using the same software. All sampling was performed in triplicate.

### Statistical analyses

A two-way ANOVA was used to compare the groups when two variables were present, namely age and time points. Tukey’s multiple comparisons test was applied to compare between different groups, of which adult vs weanling BCECs are shown for the same time point. For the viral RNA and plaque quantification, all the values were transformed to log scale and then analyses were performed (as shown in the graph). A one-way ANOVA was performed for comparison between multiple groups to compare the ability of productive viral release (PFU/ml) in ex vivo samples. In the immunofluorescence image quantification, matched comparison between adult and weanling BCECs was done. With the same viral inoculum dose, multiple t-tests were performed between 0.1 MOI adult vs 0.1 MOI weanling, 1 MOI adult vs 1 MOI weanling, and 10 MOI adult vs 10 MOI weanling. For all the graphs, mean ± standard error of the mean or mean with individual datapoints are shown.

## Results

### LACV infection-associated viral RNA is specifically abundant in BCEC microvessel isolations from weanling mice

**Fig. 1:**
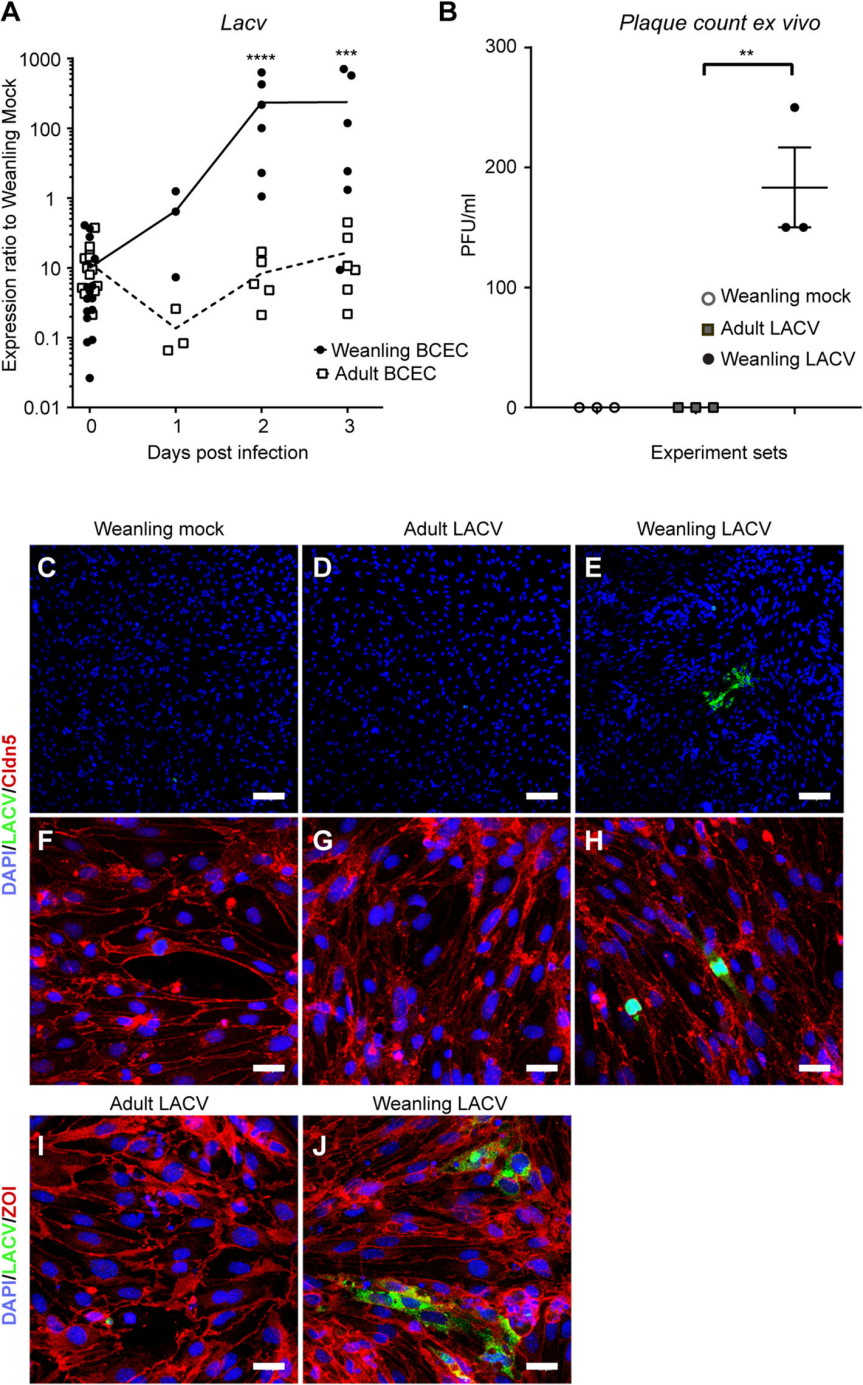
Detection of LACV in BCEC preps from weanling but not adult mice ex vivo*.* (**a**) Whole brain BCECs were isolated at 1, 2, and 3 dpi from weanling and adult LACV or mock-infected mice (N = 3 to 6 for each dpi) and then analyzed for LACV RNA. A Ttwo-way ANOVA was performed for understanding the difference in time and age. *P < 0.05, ****P < 0.0001. (**b**–**j**) Whole brain BCECs, isolated from weanling and adult LACV and mock-infected animals, were cultured until confluence. (**b**) Supernatants from LACV-infected weanling and adult BCEC were analyzed for infections virus. (*P < 0.01 between adult and weanling LACV, one-way ANOVA, I). Data are the mean from 3 samples. (**c**–**j**) Cells were immunostained with LACV (**c**–**e**) and ZO1 (**f**–**h**) or claudin 5 (cldn5, **i** and **j**). Scale bar = 100 μm (**c**–**e**) and scale bar =20 μm (**f**–**j**). Each symbol represents data obtained from a single mouse BCEC

LACV infection in weanling mice is associated with vascular leakage of the BBB, which is not observed following LACV infection in adult mice [4]. As brain capillary endothelial cells (BCECs) are an important component of the BBB, we analyzed whether there were age-related differences in BCECs, in vivo. We analyzed BCEC microvessel isolations from mice 1–3 dpi, as vascular leakage and CNS infection are first detected at 3 dpi [4]. Mocks are represented as 0 dpi (Fig. 1a). Interestingly, we found detectable viral RNA at all 3 time points in isolations from weanling mice, but not adult mice (Fig. 1a). Thus, the weanling mice microvessel isolations, enriched in BCECs, harbor viral RNA, suggesting that the microvessels and/or cells isolated with these vessels can harbor viral RNA.

### Ex vivo-isolated BCECs from infected weanling animals demonstrate presence of infectious virus in culture

As we found viral RNA in the BCECs microvessel isolations, we cultured these cells to determine if we could detect virus infection. BCECs isolated from LACV-infected weanling and adult mice at 3 dpi were cultured until confluence without changing the medium and then stained for virus infection and supernatants were analyzed for virus release. Analysis for the virus in supernatants from these cultures by plaque assay showed the presence of a low, but persistent, (~ 180 PFU/ml) level of productive viral infection in weanling BCECs. No detectable plaques were found in the culture supernatants obtained from LACV-infected adult BCECs or mock weanling BCECs (Fig. 1b). Immunohistochemistry showed that BCEC from weanling mice had small patches of cells infected with virus (green fluorescence) (Fig. 1e), which were not observed in BCECs cultures isolated from adult mice infected with LACV (Fig. 1d). Coimmunostaining showed that these LACV-infected cells were also positive for endothelial cell markers like claudin 5 and ZO1 (Fig. 1f–h and i, j). As these ex vivo BCEC microvessel isolations (Fig. 1a) and ex vivo cultures (Fig. 1b–j) were not pure BCECs and contained both cellular debris and other CNS cells, we could not distinguish whether the weanling BCECs were infected in the CNS or if the viral RNA and infectious virus were derived from another source. Regardless, these data indicate a difference in the infectability of BCECs to LACV upon long-term culture of microvessels with weanling, but not adult, BCECs showing positive-infected cells. These data suggest that weanling BCECs may be more susceptible to infection or the production of live viruses than adult BCECs.

### Cultured weanling and adult whole brain BCECs differ in their ability to produce viral plaques

**Fig. 2:**
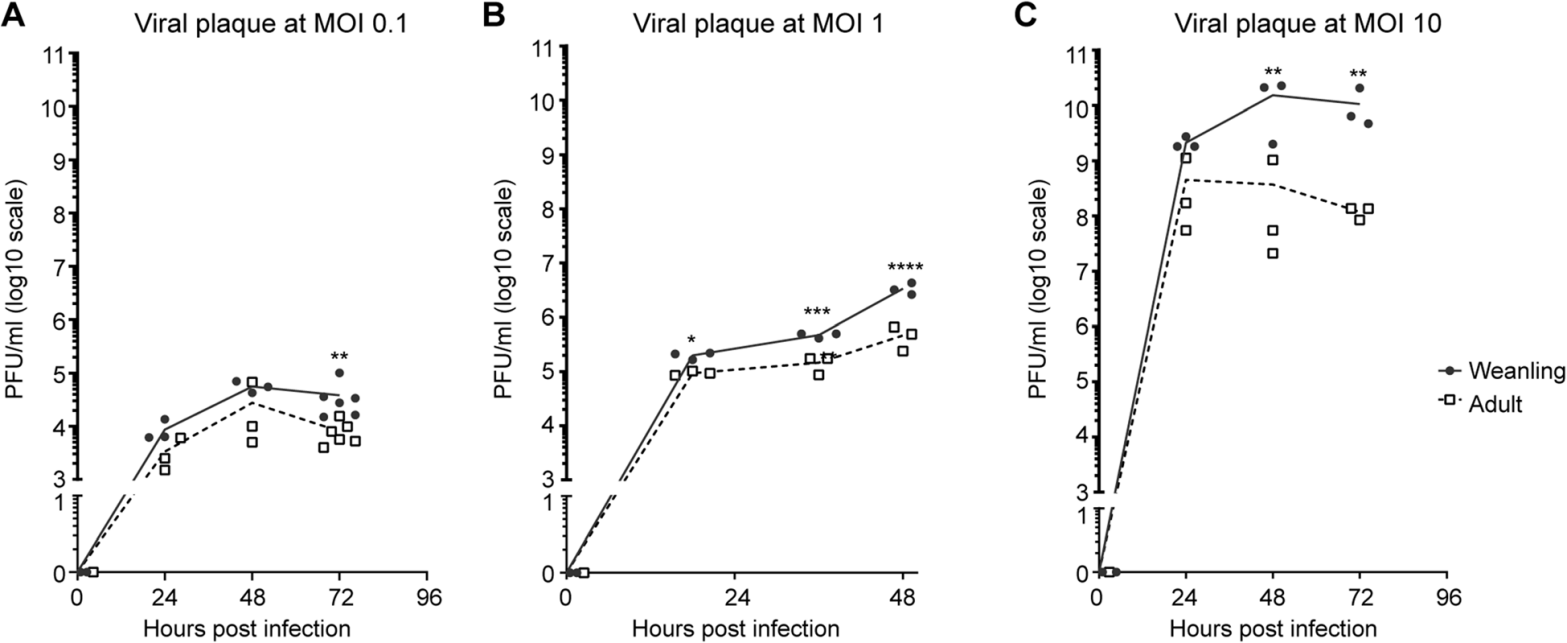
Higher infection rate of weanling BCECs compared to adult BCECs *in vitro.* Weanling and adult BCECs were isolated from uninfected mice and then cultured in vitro until cells reached confluency. Cultures were infected with LACV at (**a**) 0.1, (**b**) 1, and (**c**)10 MOI for 1 h, washed, and then incubated for indicated time points. Data are plotted as the mean obtained from 3 or more different experimental samples and all individual plaque counts are shown. A two-way ANOVA was performed for statistical analysis. *P < 0.05, **P < 0.01, ***P < 0.001, ****P < 0.0001

To directly determine if weanling BCECs differ from adult BCECs in their ability to be infected and/or allow virus production, we infected primary BCECs in vitro. BCECs were isolated from the brains of uninfected weanling and adult mice and cultured until confluence. The cultures were then infected with LACV at 0.1, 1, and 10 MOI, washed to remove the virus, and then followed for infection. We focused on the first 72 h post infection as that is a similar time frame to infection in vivo. Interestingly, PFU from culture supernatants were comparable at 24 hpi, but then were consistently higher in weanling mice at later time points (Fig. 2a–c), with up to a 2-log difference at 72 hpi in the 10 MOI cultures. Thus, in vitro, weanling and adult BCECs differ in their ability to be infected and/or produce replicating virus following infection with a similar amount of viral inoculum.

### BCECs show age-dependent difference in susceptibility to LACV infection and CPE in culture

**Fig. 3:**
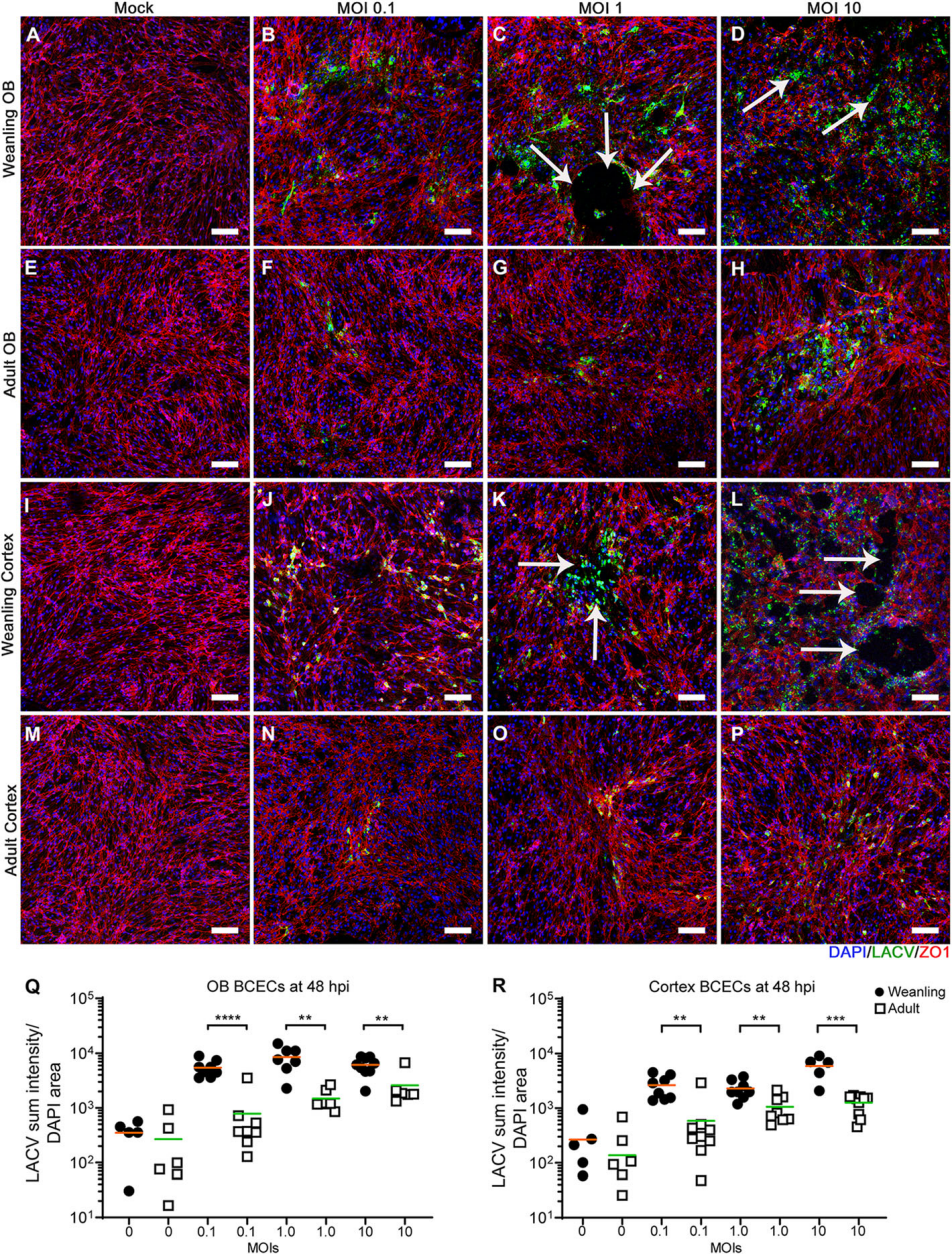
LACV infection induces higher cellular loss in weanling BCECs compared to adult BCECs. Weanling and adult BCECs, isolated separately from OB and cortex, were infected with LACV and imaged at × 10 objective magnification at 48 hpi (Scale bar = 100 μm). ZO1, LACV, and DAPI are shown in red, green, and blue channels respectively. Weanling OB BCECs (**a**–**d**) and adult OB BCECs (**e**–**h**) were mock-infected (**a**, **e**) or infected with LACV at MOIs of 0.1(**b**, **f**), 1 (**c**, **g**), and 10 (**d**, **h**). Weanling cortical BCECs (**i**–**l**) and adult cortical BCECs (**m**–**p**) were mock-infected (**i**, **m**) or infected with LACV at MOIs of 0.1(**j**, **n**), 1(**k**, **o**), and 10 (**l**, **p**). All images are representative of multiple areas from wells of two experiments (a single experiment set was used for setting the background fluorescence and image quantification). Arrows show areas of viral infection and loss of cells. Quantification of sum intensity of LACV to DAPI (**q**, **r**) area using several randomized images from each set. Quantification of each image is shown as a single symbol along with the mean (orange bar: mean of weanlings and green bar: mean of adults) for each group. For each viral inoculum dose, multiple unpaired t-tests were performed between weanling OB vs adult OB and weanling cortical vs adult cortical BCECs. *P < 0.05, **P < 0.01, ***P < 0.001, ****P < 0.0001

**Fig. 4:**
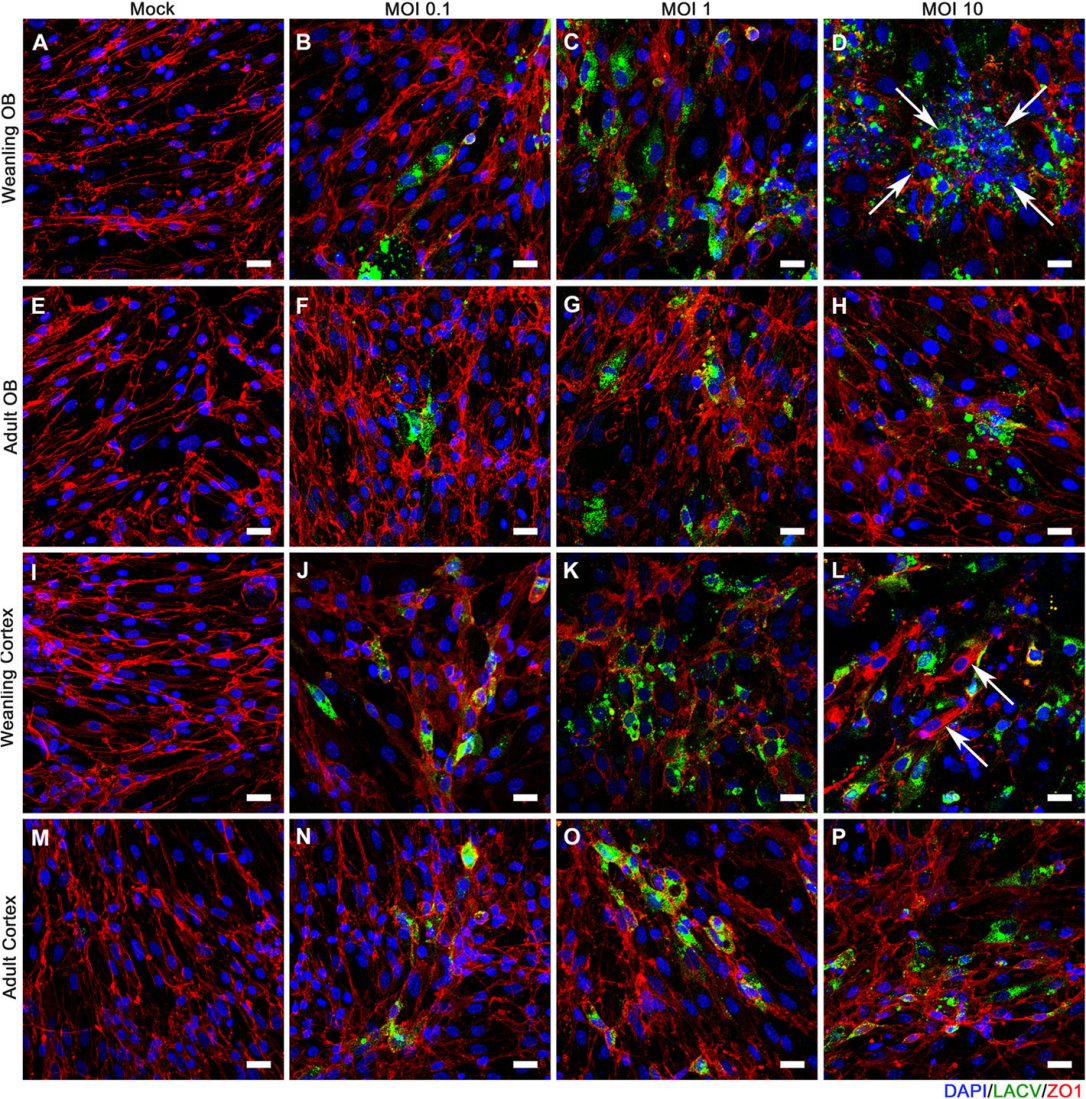
LACV infection induced cellular morphology changes in BCECs. Cultures described in Fig. 3 were stained for DAPI (blue), LACV (green), and ZO1 (red) and imaged at × 40 objective magnification (Scale bar = 20 μm) to examine morphological changes of infected cells. Weanling OB BCECs (**a**–**d**) and adult OB BCECs (**e**–**h**) are shown. OB BCECs were mock-infected (**a**, **e**) or infected with LACV at MOIs of 0.1 (**b**, **f**), 1 (**c**, **g**), and 10 (**d**, **h**). Weanling cortical BCECs (**i**–**l**) and adult cortical BCECs (**m**–**p**) were mock-infected (**i**, **m**) or infected with LACV at MOIs of 0.1 (**j**, **n**), 1 (**k**, **o**), and 10 (**l**, **p**). Cell aggregation and disruption of ZO1 staining are shown by the arrow (**d**). Localization of ZO1 in an intracellular compartment is shown by an arrow (**l**). Combined counts of images from OB and cortex samples at 10 MOI inoculum doses of LACV and ZO1 stained samples showed an average of 3.7 ± 0.5 foci of cellular loss from 17 representative images of weanling BCECs. In adult BCEC, an average of 0.6 ± 0.3 foci of cellular loss were observed from 10 representative images. 0.2 ± 0.1 syncytia were observed in a total of 13 images of weanling BCECs whereas no syncytium was seen in a total of 12 images of adult BCECs

In previous studies, BCECs located in the OB region of weanling brains were more susceptible to LACV-induced vascular leakage than BCECs in the cortex [4]. Thus, we hypothesized that BCECs from the OB region of weanling mice may have different responses to in vitro infection than those from the cortex. BCECs from the OB (Figs. 3 and 4a–h) and cortical regions (Figs. 3 and 4i–p) of the brain from weanling (Figs. 3 and 4a–d and i–l) or adult mice (Fig. 3 and 4e–h and m–p) were cultured, kept uninfected as mock samples, and infected separately with 0.1, 1, and 10 MOI of LACV and then analyzed for infection using markers against LACV (green) to detect virus and ZO1 (red) to look at endothelial cell structure in the culture. As expected, cultures infected with higher MOIs had more infected cells compared to low MOIs (Fig. 3). At a lower magnification, OB and cortical BCECs from weanling mice (arrow, Fig. 3d and l), had widespread viral infection. Infection was associated with loss of BCECs from culture and cellular aggregation in a few areas (Fig. 3d, l, white arrows). Some of these cytopathic effects (CPEs) denoted by cellular aggregation, loss of ZO1, and loss of cells were also observed in weanling OB BCECs infected at 1 MOI (Fig. 3c, k, white arrows). Adult BCECs from the OB (Fig. 3f–h) and cortex (Fig. 3n–p) were comparatively resistant to LACV  virus infection and/or replication, as demonstrated by a lack of detectable CPE and lower viral spread in those samples. Analysis of random fields from all the experimental groups demonstrated significant differences between weanling BCECs and adult BCECs for all MOIs, but no difference between OB and cortex BCECs (Fig. 3q–r). Higher magnification images of these cultures showed aggregation of infected cells (Fig. 4d) and disruption of ZO1 in weanling OB and cortex BCECs (arrow, Fig. 4p). At 24 hpi, OB BCECs from weanling mice started to lose their cell-to-cell contacts and weanling BCECs had the most abundant viral infection. At this point, prominent virus infection was observed for both adult and weanling BCECs (supplementary figure 1 and 2). This infection pattern became gradually more restricted in adult BCECs at 48 and 72 hpi. However, in weanling cultures, virus-induced morphological changes like cellular aggregation, loss of cell junction adaptor protein ZO1 and caspase-3-mediated cellular apoptosis became more evident at those later time points. LACV infection was comparatively reduced in the weanling OB BCECs due to prominent cell loss at 72 hpi (supplementary figure 3D and 4D). Thus, in vitro cultures of BCECs from weanling mice had a higher level of infected cells compared to BCECs from adult mice, which was associated with loss of cells and altered cellular morphology. A 0.5-log difference between the weanling OB and cortical BCECs was also observed at 1 MOI only but not at 0.1 or 10 MOI, suggesting that regional differences in BCECs for LACV infection, in vitro, were probably dose dependent. In contrast, age-related differences in virus infection were observed at all doses.

### Caspase-3-dependent cell death is observed in LACV-infected weanling BCECs

**Fig. 5:**
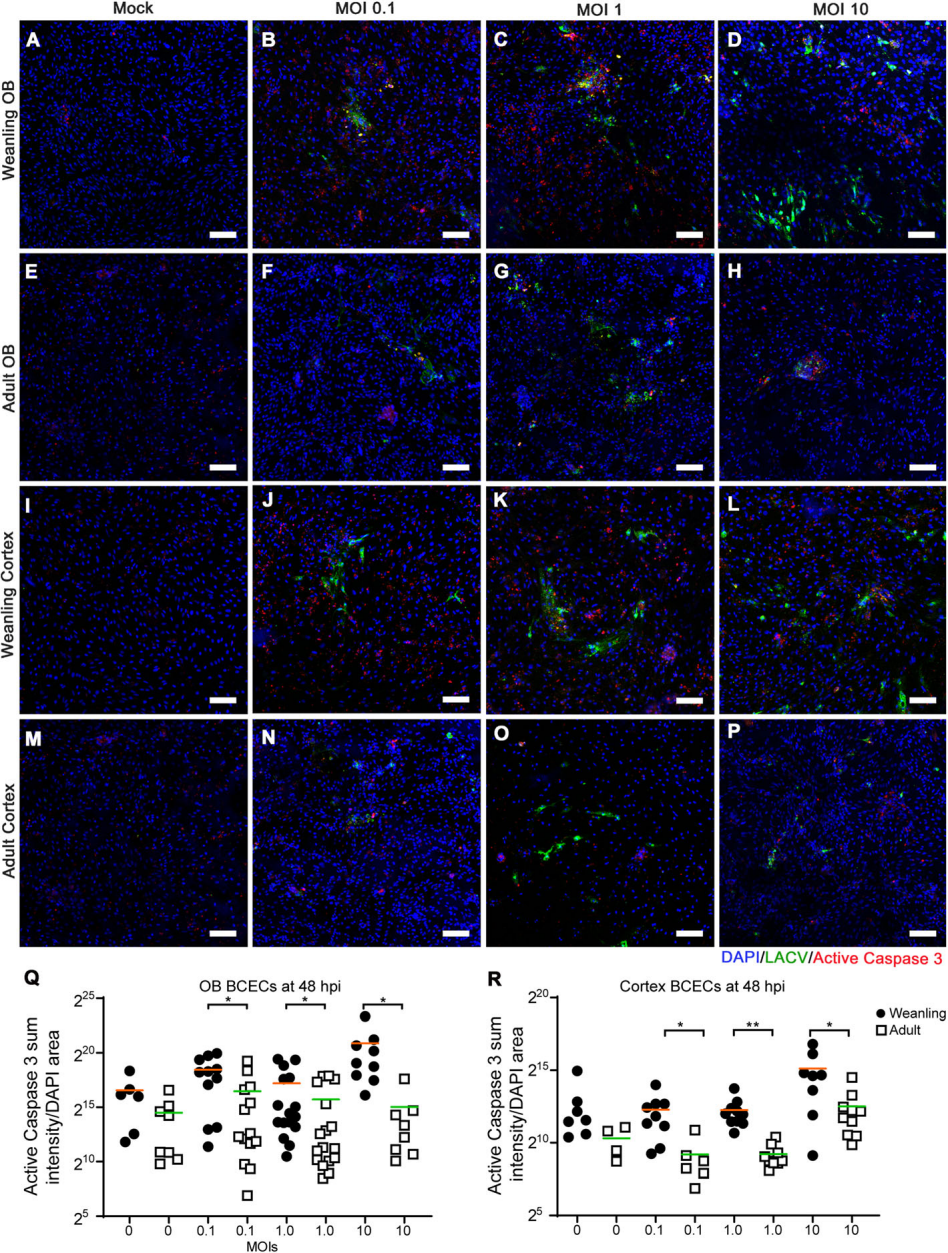
CPE observed in weanling BCECs associated with active caspase-3. Weanling and adult BCECs isolated from OB and cortex were infected with LACV. At 48 hpi, the samples were stained for LACV (green), active caspase-3 (red), and nuclear counterstain DAPI (blue). Scale bar represents 100 μm. BCECs were mock-infected (**a**, **e**, **i**, **m**) or infected with LACV with an MOI of 0.1 (**b**, **f**, **j**, **n**), 1 (**c**, **g**, **k**, **o**), and 10 (**d**, **h**, **l** and **p**). Weanling OB (**a**–**d**), adult OB weanling cortical (**e**–**h**), weanling coritcal (**i**–**l**), and adult cortical (**m**–**p**) BCECs are shown. Arrows indicate active caspase-3 staining in and around LACV-infected cells (**b**, **c**, **d**, **j**, **k**, **l**) or regions of cell loss (**d**). Relative active caspase-3 presence per cell was quantified by calculating the active caspase-3 sum intensity-to-DAPI area ratio from several randomized from a single experiment set that was used for setting the background fluorescence and image quantification of all group. Quantification of each datapoint is shown as a single symbol along with the mean (orange bar: mean of weanlings and green bar: mean of adults) for each group (**q**, **r**). Multiple unpaired t-tests were performed for statistical analyses between matched groups and significance is represented by *P < 0.05 and **P < 0.01

**Fig. 6:**
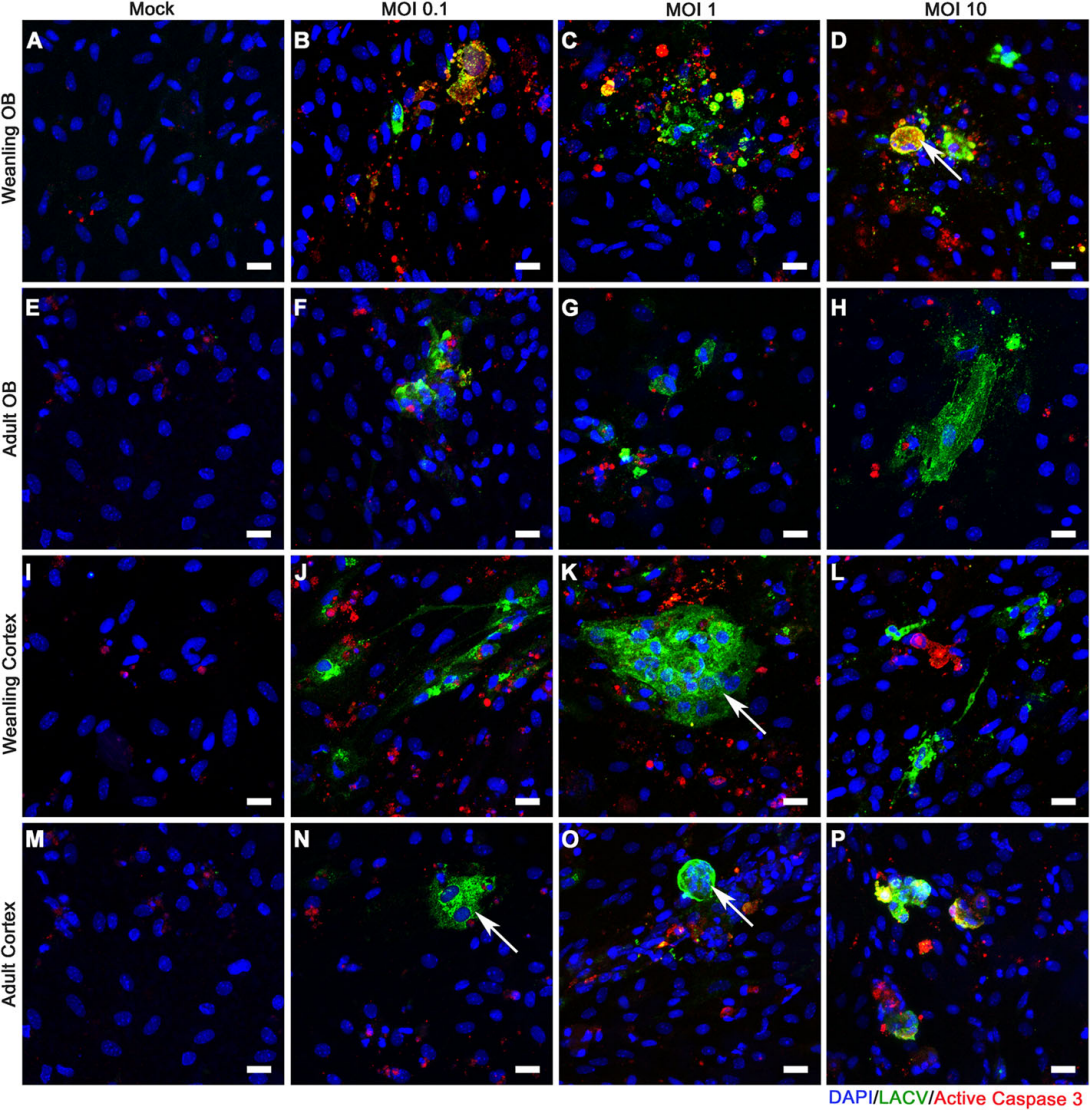
LACV infection induces syncytium-like aggregation and bystander cell death. Cultures described in Fig. 5 were stained for DAPI (blue), LACV (green), and active caspase-3 (red) and imaged using a × 40 objective. Weanling OB BCECs (**a**–**d**), adult OB BCECs (**e**–**h**) were mock-infected (**a**, **e**) or infected with LACV at MOIs of 0.1 (**b**, **f**), 1 (**c**, **g**), and 10 (**d**, **h**). Weanling cortical BCECs (**i**–**l**) and adult cortical BCECs (**m**–**p**) were mock-infected (**i**, **m**) or infected with LACV at MOIs of 0.1 (**j**, **n**), 1 (**k**, **o**), and 10 (**l**, **p**). Large, syncytium-like aggregation is noted as arrows in **d** and **k**). Combined counts of images from the OB and cortex samples at 10 MOI inoculum doses of LACV and active caspase-3-stained samples demonstrated an average of 2.3 ± 0.7 of cellular loss were counted per image of 10 representative images from weanling BCECs. In adults, 0.9 ± 0.3 foci of cellular loss were observed per image from10 representative images. 0.6 ± 0.2 syncytia were observed in a total of 11 images from weanling BCECs. In adults, 0.36 ± 0.28 syncytia were counted per image from 11 representative images. All images were at 10 MOI of inoculation dose. Scale bar = 20 μm

Previous studies demonstrated that LACV can induce cell death through a caspase-3-dependent mechanism [29]. Therefore, infected cultures were stained for active caspase-3 (red) and LACV (green) at 48 hpi. Active caspase-3-positive cells were found in regions of virus infection in weanling BCECs cultures (Fig. 5b–d, j–l). Arrows indicate areas of CPE. Higher magnification analysis of LACV-infected BCECs showed several areas of robust apoptosis in areas of virus infection, although some active caspase-3-positive apoptotic cells were not infected, and some infected cells were not apoptotic. Additionally, occasional formations of syncytium-like aggregations of infected cells were observed in cultures (arrow, Fig. 6d, k, n, o), although all these cells were not always apoptotic. Mock-inoculated cells also showed spontaneous presence of active caspase-3 around some cell clusters (Fig. 6a, e, i, and m). For both OB and cortical cultures, BCECs isolated from weanling mice (Figs. 5 and 6b–d and j–l) had higher amount of caspase-3-positive stain compared to adults (Figs. 5 and 6f–h and n–p). In addition, weanling OB BCECs (Fig. 6b–d) had a higher basal level of active caspase-3 expression compared to the cortical BCECs (Fig. 6j–l). Upon infection with LACV, the expression of active caspase-3 was increased substantially and included both LACV-infected and LACV-uninfected cells. Thus, OB BCECs had a higher level of basal and virus-induced apoptosis compared to the cortical BCECs (Fig. 5q, r).

The increase in active caspase-3 cells was not observed in adult BCECs cultures at any MOI (Fig. 5q, r), suggesting that weanling BCECs were more susceptible to LACV-induced apoptosis than adult BCECs. As many of these caspase-3 positive cells were uninfected in the weanling BCEC cultures, a bystander effect may be responsible for the apoptotic death of these cells rather than a direct effect of virus infection. To better quantify the difference in LACV-induced apoptosis between weanling and adult BCECs, we analyzed cultures over the course of infection with Cytotox Green dye. Cells were measured every 3 h over a 72-h period post infection. Similar results were observed with a significant difference in the incorporation of Cytotox Green dye in LACV-infected weanling BCECs compared to mock-infected weanling BCECs or LACV-infected adult BCECs (Supplementary figure 0^5^ and Video 1 and 2, N = 3). Thus, LACV infection of weanling BCEC cultures resulted in increased apoptosis, including non-infected cells suggesting that infection can lead to bystander cell death.


**Additional file 2.**


**Additional file 3.**

### Type I IFN responses were not elevated in adult BCECs

One potential mechanism by which BCECs from adult mice may be more resistant to virus infection and replication would be a stronger type I IFN response. Therefore, we analyzed mRNA expression of type I IFN genes interferon α4 (*Ifna4*), interferon β (*Ifnb*), and interferon responsive factor 7 (*Irf7*) in brain tissue from LACV-infected adult and weanling mice, ex vivo-isolated BCECs as well as *Ifna4*, *Ifnb*, and interferon-induced protein with tetratricopeptide repeats 1 (*Ifit1*) expression in vitro infected BCECs from adult and weanling. Interestingly, all three type I IFN-related genes had higher mRNA expression in weanling mice and BCECs compared to adults (supplementary figure 6). Thus, the susceptibility of weanling BCECs to LACV infection and damage does not appear to be due to a weak type I IFN response.

### Detection of morphological changes in LACV-infected weanling mice brain capillaries and CNS parenchyma by electron microscopic analysis

**Fig. 7:**
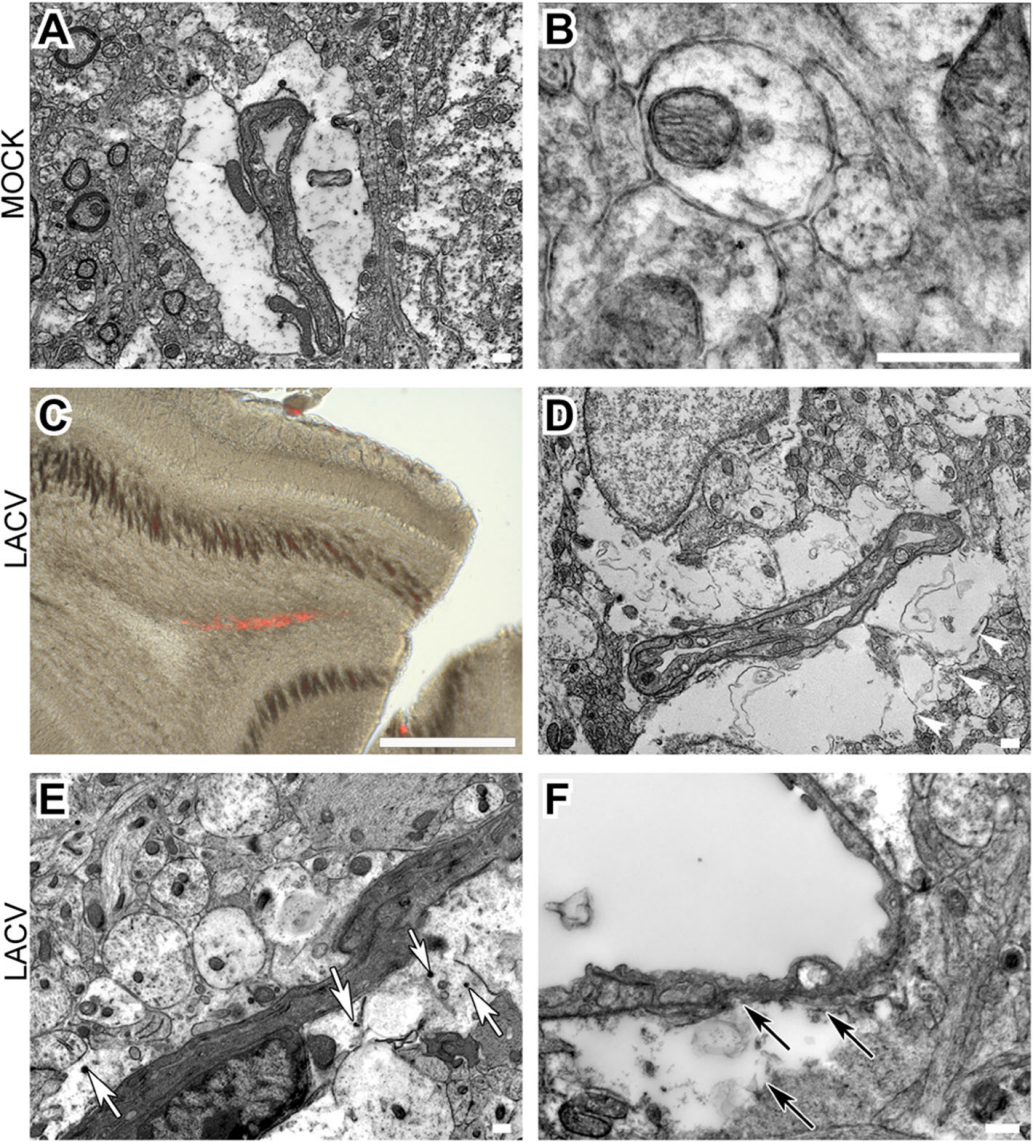
Alteration of cerebrovascular tissue morphology in LACV induced vascular leakage. CLEM images of vibratome sectioned OBs from mock (**a**, **b**) and LACV-infected mice (**c**–**f**) injected with 100-nm fluorescent beads at 3 dpi. BCEC ultrastructure from mock-infected OB (**a**, **b**). LM image of vibratome-sectioned LACV-infected mice OB used to identify vascular leakage in blood vessels by TEM (**c**), with beads in brain parenchyma shown with white arrows (**e**). Ultrastructure of BCEC from areas of vascular leakage (**d**, **f**) showing compromised perivascular tissue region indicated by white arrowheads and altered BCEC morphology indicated by black arrows. TEM scale bar = 500nm, LM scale bar = 500 μm

In order to determine whether some of the morphological changes (reduction of cell-to-cell contact and CPE) that we observed in BCEC in vitro were also observed in vivo, we examined BCECs in the CNS of weanling mice. Weanling mice infected with LACV were injected IV with virus-sized fluorescent beads at 3 dpi. Brain tissue from these mice were then analyzed by correlative light and electron microscopy (CLEM) technique to identify areas in the brain with associated vascular leakage. Specifically, areas of vascular leakage in the brain of infected mice was detected in vibratome sections by the presence of fluorescent beads. Vascular leakage was not observed in uninfected mice. In LACV-infected mice, fluorescent beads were found in the OB region, representing a region of interest (ROI) for TEM analysis. Tissue from this region was then examined by TEM for ultrastructural differences using similar regions from mock-infected mice as controls. In the mock-infected mice, some BCECs in this region showed large perivascular space/less dense astrocytic end feet (Fig. 7a), while others showed normal NVU structure. However, even in the BCECs with large perivascular space, higher magnification of the organellar structure surrounding the NVU looked uncompromised (Fig. 7b). Some of the BCECs present in the OB region of LACV-infected mice had similar large perivascular space. Panel C represents an ROI for TEM analysis, where red fluorescence denotes area of vascular leakage inside the OB region of LACV-infected mice. However, in comparison to mock-inoculated mice, the BCEC in infected mice had poor structural integrity of perivascular tissue region and endfeet structure (white arrowheads, Fig. 7d). Evidence of vascular leakage was observed by the presence of multiple beads in the parenchyma (white arrow, Fig. 7e). Importantly, the basement membrane structures were damaged in the BCECs in this region (black arrow, Fig. 7f), showing that the morphological features of NVU were compromised. Although no LACV particles were observed in the vicinity of the beads or surrounding BCEC, the compromised NVU was only observed in infected mice. Thus, there is detectable damage to the NVU during LACV infection in weanling mice to allow virus-sized particles inside brain parenchyma.

## Discussion

Age-related susceptibility to LACV-encephalitis in mice is dependent, in large part, on the ability of LACV to invade the CNS of young mice. In the current study, we found that weanling BCECs are more susceptible to LACV infection and virus-induced cell death than adult BCECs. Ex vivo culture of BCECs from infected mice showed infection of weanling BCECs, but not adult BCECs. This difference could be due to differences in circulating virus that reaches the brain capillary endothelial cells, as weanling mice have higher levels of peripheral virus [2]. Therefore, we also examined infection in vitro with identical virus doses. In vitro, both adult and weanling BCECs were infected with LACV; however, weanling BCECs had a statistically significant higher number of infected cells. This higher level of infection was associated with areas of BCEC loss and altered ZO1 localization within the cell and promoted an increase in apoptotic BCECs, as detected by caspase-3 staining. Thus, one of the reasons by which young mice may be more susceptible to LACV encephalitis compared to adult mice may be the difference in their response to LACV exposure.

In previous studies, we did not observe any obvious infection of microvessels in LACV-infected weanling mice by IHC analysis of brain tissue from weanling mice or by analysis for viral proteins in BCECs directly ex vivo [4]. In contrast, the BCECs isolated from infected weanling animals and cultured showed small areas of infection and a productive number of viral plaques upon culturing ex vivo-isolated BCECs. The discrepancy between the lack of infection in vivo versus the detection of infected cells ex vivo could be due to several factors. First, BCECs may be infected in vivo, but at such a low percentage to be undetectable by IHC or direct ex vivo analysis without amplification of several days in culture. The detection of the virus after multiple days of culture, but not directly ex vivo, could also indicate that BCECs are not productively infected in vivo, but are instead trafficked virus across the BBB, with virus replication only occurring when cells were actively cultured in vitro. Finally, as these cultures were not sorted for endothelial cells, they would initially have other CNS cell types and/or exosomes/cellular debris carrying virus particles that could subsequently infect endothelial cells in vitro. Although these differences would be difficult to parse out, they do indicate that productive infection of endothelial cells by LACV in vivo, if occurring, would be a very rare event.

The lack of productive infection in vivo does not indicate that the BCECs are not impacted by LACV. Indeed, vascular leakage of brain capillaries is an important mechanism by which LACV invades the CNS. The CLEM analysis of the sites of vascular leakage showed low structural integrity around the perivascular tissue region and end feet structures of brain capillaries in vivo, in the absence of virus particles. In vitro, virus exposure led to caspase-3-dependent apoptosis of both infected and bystander uninfected BCECs from young animals. Thus, the important impact of LACV on BCECs may not be direct infection but rather the response of uninfected BCECs to LACV. The increase in apoptosis and the morphological changes that were observed in both infected and bystander, uninfected BCECs from weanling mice in this study support the fact that LACV can have negative consequences on BCECs even in the absence of infection. In addition, we also observed a higher basal level of active caspase-3 expression in the weanling OB BCECs, which is further enhanced by LACV infection. This suggests that the weanling BCECs in the OB region are highly susceptible to LACV infection as well as infection-induced bystander cell death in vitro, which may actively contribute to the destruction of BCECs and induce vascular leakage as observed in vivo.

LACV also induced alteration of ZO1 expression in primary cultured BCECs obtained from uninfected weanling mice and infected in vitro. ZO1 is reported to regulate endothelial AJs, control angiogenesis and barrier formation. ZO1 performs as a major cytoskeletal organizer in ECs, and controls the overall distribution of F-actin. The previous reports demonstrated that LACV entry might be dependent on cytoskeletal rearrangement [4]. Virus infection in endothelial cells can cause alteration of ZO1 expression or surface localization [30–32]. Following LACV infection in vitro, a few of the BCECs exhibit cytoplasmic ZO1 staining. Alteration of ZO1 localization might alter permissibility and induce cytoskeletal remodeling in the weanling BCECs. This, in turn, may induce the leakiness of BBB and facilitate viral transport across the BCECs. In addition, cellular aggregation was observed around heavily infected areas, where ZO1 staining was also depleted. Altogether, altered localization and depletion of ZO1 staining in weanling BCECs might suggest a LACV-induced disruption of barrier function in the weanling BCECs.

Another defining feature of LACV infection of BCECs from weanling mice was the “syncytium-like aggregation” of some BCEC cells. These syncytia suggest the potential for viral spread by cell-to-cell fusion, which can be very difficult to detect in vivo. Viruses like MHV-A59 induce syncytium formation, which in turn, can be associated with cytoskeletal and organellar rearrangements [33]. In addition, virus-induced syncytium formation can lead to infected cell death [34]. Thus, the in vitro culture of BCECs provides a model to study the LACV-induced syncytium-like aggregation formation, induction of CPE, and disruption of ZO1. LACV infection-induced syncytium formation correlates with the cytoskeletal rearrangements observed in ex vivo-isolated cells [4]. Gaining a better understanding of why weanling BCECs undergo “syncytium-like aggregation,” while adult BCECs generally do not, may provide insight into the morphological changes associated with weanling BCECs, in vivo, during virus infection.

In the BCEC culture, the OB and cortical BCECs also show differences in the spread of LACV. Although this difference in infectivity was not observed with all inoculum doses, OB BCECs were more apoptotic than adult BCECs. This is also consistent with a previous report [4] showing that OB BCECs are primary sites of viral entry, where higher cell death creates a site of vascular leakage and viral entry. Previously, it has also been reported that the OB BCECs are more susceptible to LACV infection-induced viral leakage in vivo [4]. The prominent infection of weanling BCECs from both the OB and cortical region might be due to partial loss of spatial properties including density and three-dimensional arrangement of blood vessels in the OB/olfactory tract as well as the loss of basolateral polarization of the cells. The weanling and adult BCECs also show different characteristics of viral infection spread and CPE at different timepoints. Although both adult and weanling BCECs are infected in culture, the adult BCECs are capable of restricting virus infection, infection-associated morphological changes, and CPE while weanling BCECs are not. The strong effect on bystander uninfected BCECs of weanling mice may explain how LACV can induce vascular leakage in brain capillaries in the absence of detectable infection in vivo.

Interestingly, protection against virus infection in adult BCEC did not correlate with stronger type I IFN responses by these cells. Instead, type I IFN responses were higher in weanling brain tissue from weanling mice as well as both ex vivo- and in vitro-stimulated BCECs. This type I IFN response correlates most strongly with virus levels both in vivo and in vitro, suggesting that the type I IFN response is more a response to virus infection and not responsible for protection from LACV infection. Further investigation into the gene and protein expression that differ between BCECs from weanling versus adult mice may reveal whether there are specific genes or a subgroup of genes that are age dependent and that influence BCEC responses to virus infection.

Along with age-related factors that control BCEC infection, alteration of ZO1, syncytium formation in vitro, and cell death, the EC morphology is altered throughout developmental stages. The perivascular spaces surrounding BCECs become smaller, BCEC lining becomes thinner during development and the adult junctions are mostly closed throughout their length [35]. In 5-day-old animals, the endothelial barrier is highly permeable, demonstrated by increased leakage of a tracer around the ECs [36]. In our study, we observed that uninfected weanling mice can have a large perivascular space, but no leakage of virus-sized particles was observed. However, with LACV infection, we observed some of the BCECs have an altered morphology in and around the NVU and some of the virus-sized fluorescent beads (~ 100 nm) were localized in the brain parenchyma. Thus, some of the same alterations of BCEC barrier function found by culturing and infecting weanling BCECs in vitro are reflected in vivo and correlated to LACV-induced vascular leakage.

## Conclusion

This study demonstrates that age can be a determining factor in how BCECs respond to virus infection. Indeed, we found that younger BCECs were more susceptible to LACV infection and apoptosis, even when cultured ex vivo. The age-related susceptibility of BCECs to virus-induced apoptosis occurred in both uninfected and infected cells, correlating with the ability of the virus to induce damage without clear signs of infection in vivo. Furthermore, CLEM analysis showed virus-induced damage to brain vasculature sufficient to allow virus-sized particles to enter the CNS of weanling mice. Future studies directed at determining how young versus more mature BCECs differ in their response to virus infection will be key in understanding the mechanisms of virus entry in the CNS as well as developing therapeutics to prevent BBB damage.

## Supplementary Information

**Additional file 1.**

## Data Availability

The datasets used and/or analyzed during the current study are available from the corresponding author on reasonable request.

## Abbreviations

AJ: Adherens junction
BBB: Blood-brain barrier
BCECs: Brain capillary endothelial cells
CPE: Cytopathic effects
CLEM: Correlative light electron microscopy
Cldn5: Claudin 5
CNS: Central nervous system
DAPI: 4′,6-Diamidino-2-phenylindole
DMEM: Dulbecco’s modified Eagle’s medium
dpi: Days post infection
Hpi: Hours post infection
GAPDH: Glyceraldehyde 3-phosphate dehydrogenase
GJ: Gap junction
i.p.: Intraperitoneal
i.c.: Intracranial
IHC: Immunohistochemistry
LACV: La Crosse virus
MOI: Multiplicity of infection
NVU: Neurovascular unit
OB/OT: Olfactory bulb/tract
PBS: Phosphate-buffered saline
PFU: Plaque forming unit
PFA: Paraformaldehyde
ROI: Region of interest
TJ: Tight junction
TEM: Transmission electron microscopy
ZO1: Zona occludens
