# Supplementary material for: Age influences susceptibility of brain capillary endothelial cells to La Crosse virus infection and cell death
Source: J Neuroinflammation. 2021 Jun 3;18:125. doi: 10.1186/s12974-021-02173-4 (PMC8173794; doi:10.1186/s12974-021-02173-4)
Supplement: Supplementary file 3 — Additional file 1. [file 12974_2021_2173_MOESM1_ESM.pdf]

Additional figures and figure legends

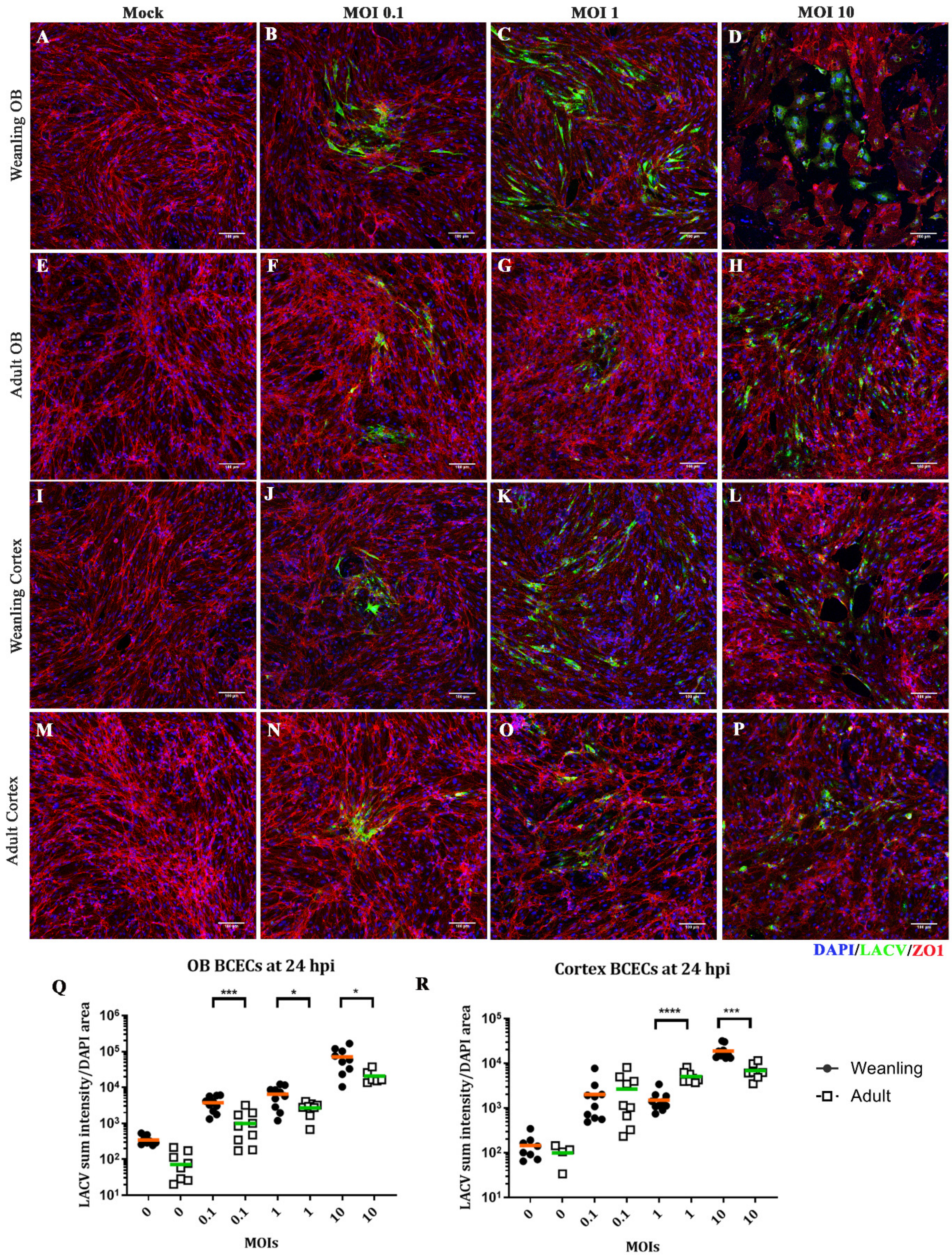

Supplementary fig. 1 LACV infection and associated cytopathic changes observed at 24 hpi at 10X objective magnification

Cells were infected with LACV until 24 hpi and then stained with ZO1 (red), LACV (green) and nuclear counterstain DAPI (blue). *In vitro* cultured weanling and adult BCECs were infected at 10 (D, H, L and P), 1 (C, G, K and O) and 0.1 MOI (B, F, J and N). Mock infected cells were maintained in parallel (A, E, I and M). Weanling OB (A-D), weanling cortical (I-L), adult OB (E-H) and adult cortical (M-P) BCEC cultures are shown. For all the images, scale bar = 100 μm. Quantitative comparison of weanling and adult BCECs from OB (Q) and cortical (R) region respectively (orange bar: mean of weanlings and green bar: mean of adults). Multiple t-tests for each MOI showed weanling BCECs had a higher viral staining than adult BCECs (*$P<0.05$, ** $P<0.01$, *** $P<0.001$, **** $P<0.0001$).

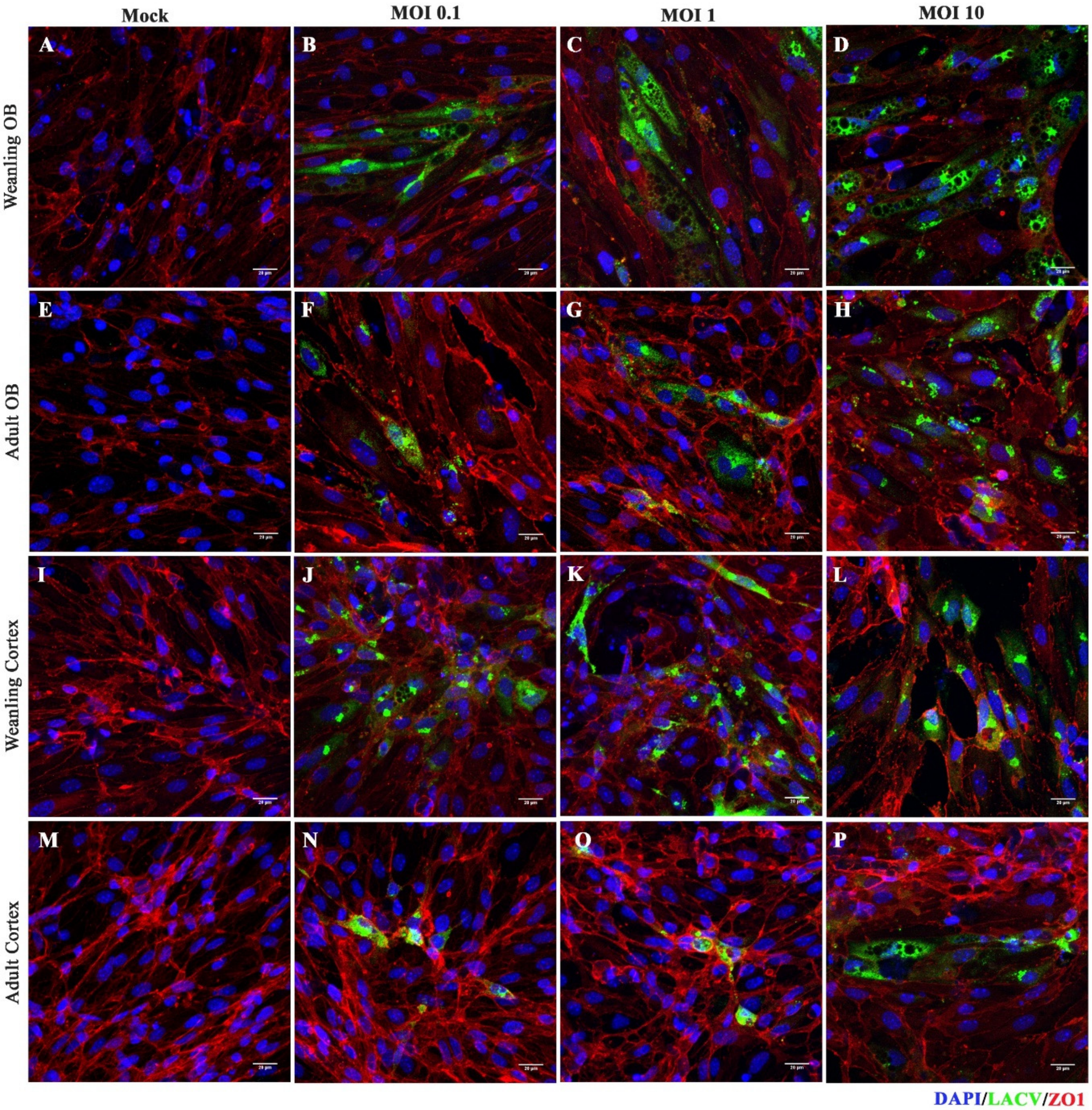
Mock
MOI 0.1
MOI 1
MOI 10
Weanling OB
Adult OB
Weanling Cortex
Adult Cortex
A
B
C
D
E
F
G
H
I
J
K
L
M
N
O
P
DAPI/LACV/ZO1

Supplementary fig. 2 LACV infection induced cellular morphology changes observed at 24 hpi at 40X objective magnification

Higher magnification images (using 40X objective) were obtained from adult and weanling BCECs at 24 hpi, as described in supplementary image 1. Cells were stained with ZO1 (red), LACV (green) and nuclear counterstain DAPI (blue). Weanling OB BCECs (A-D), adult OB BCECs (E-H) were mock-infected (A, E) or infected with LACV at MOIs of 0.1 (B, F), 1 (C, G) and 10 (D, H). Weanling cortical BCECs (I-L) and adult cortical BCECs (M-P) were mock-infected (I, M) or infected with LACV at MOIs of 0.1 (J, N), 1 (K, O), and 10 (L, P). Scale bar = 20 μm.

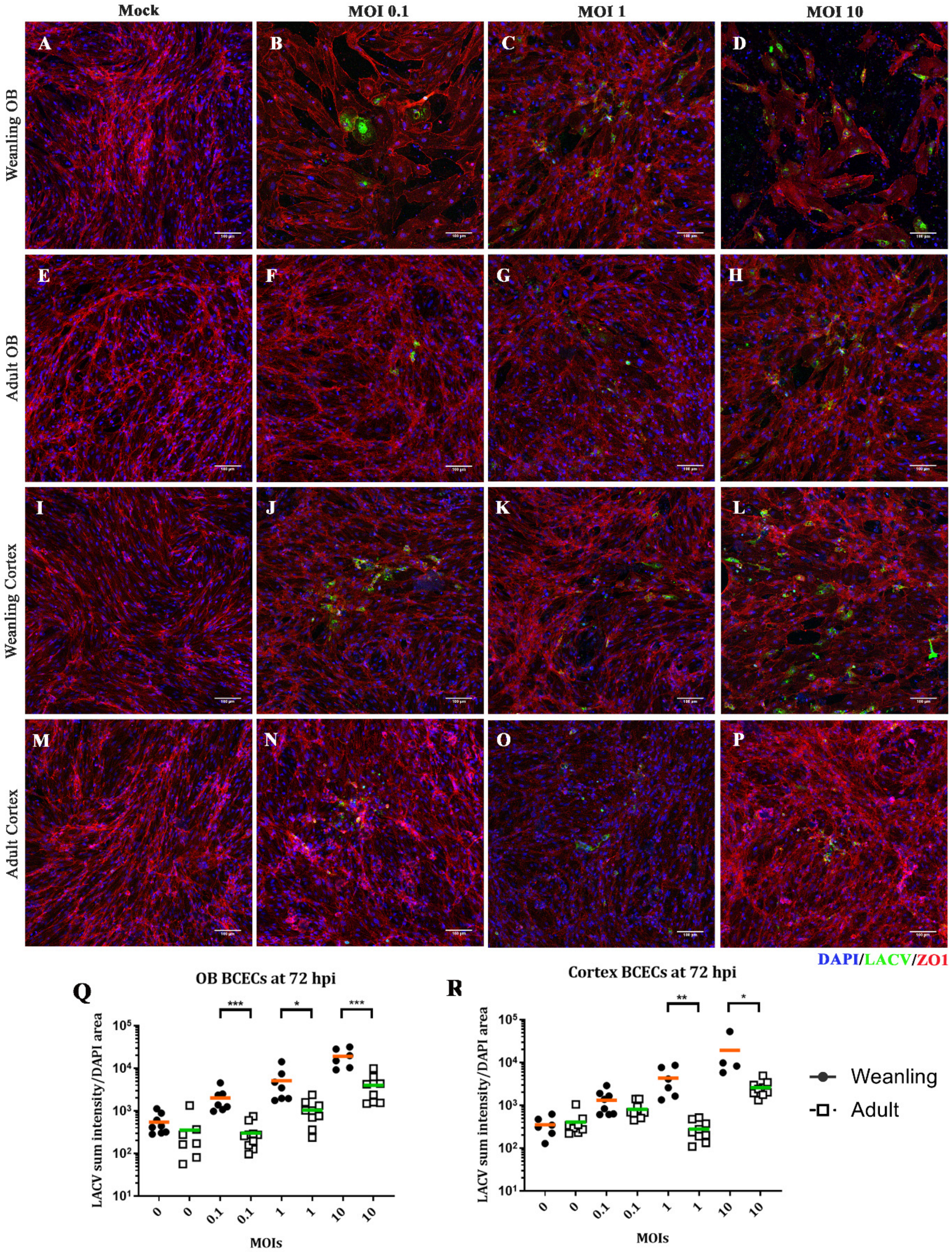

Mock
MOI 0.1
MOI 1
MOI 10
Weanling OB
Adult OB
Weanling Cortex
Adult Cortex
A
B
C
D
E
F
G
H
I
J
K
L
M
N
O
P
DAPI/LACV/ZO1
Q
OB BCECs at 72 hpi
R
Cortex BCECs at 72 hpi
LACV sum intensity/DAPI area
MOIs
Weanling
Adult

Supplementary fig. 3 Loss of weanling BCECs at 72 hpi after LACV infection observed at 10X objective magnification

Weanling and adult BCECs were infected with LACV for 72 hours and stained for ZO1 (red) and LACV (green) and DAPI (blue). BCECs were mock-infected (A, E, I, M) or LACV infected cells at an MOI of 0.1 (B, F, J, N), 1 (C, G, K, O) and 10 (D, H, L and P). BCECs were isolated from OB and cortex, from weanling as well as adult mice and are represented as weanling OB (A-D), adult OB (E-H), weanling cortical (I-L) and adult cortical (M-P) BCECs. Scale bar represents 100 μm. Panel Q and R demonstrates the comparison weanling and adult BCECs (orange bar: mean of weanlings and green bar: mean of adults, all individual datapoints are shown). Multiple t-tests showed weanling BCECs had a higher viral staining than adult BCECs (*$P<0.05$, ** $P<0.01$, *** $P<0.001$, **** $P<0.0001$).

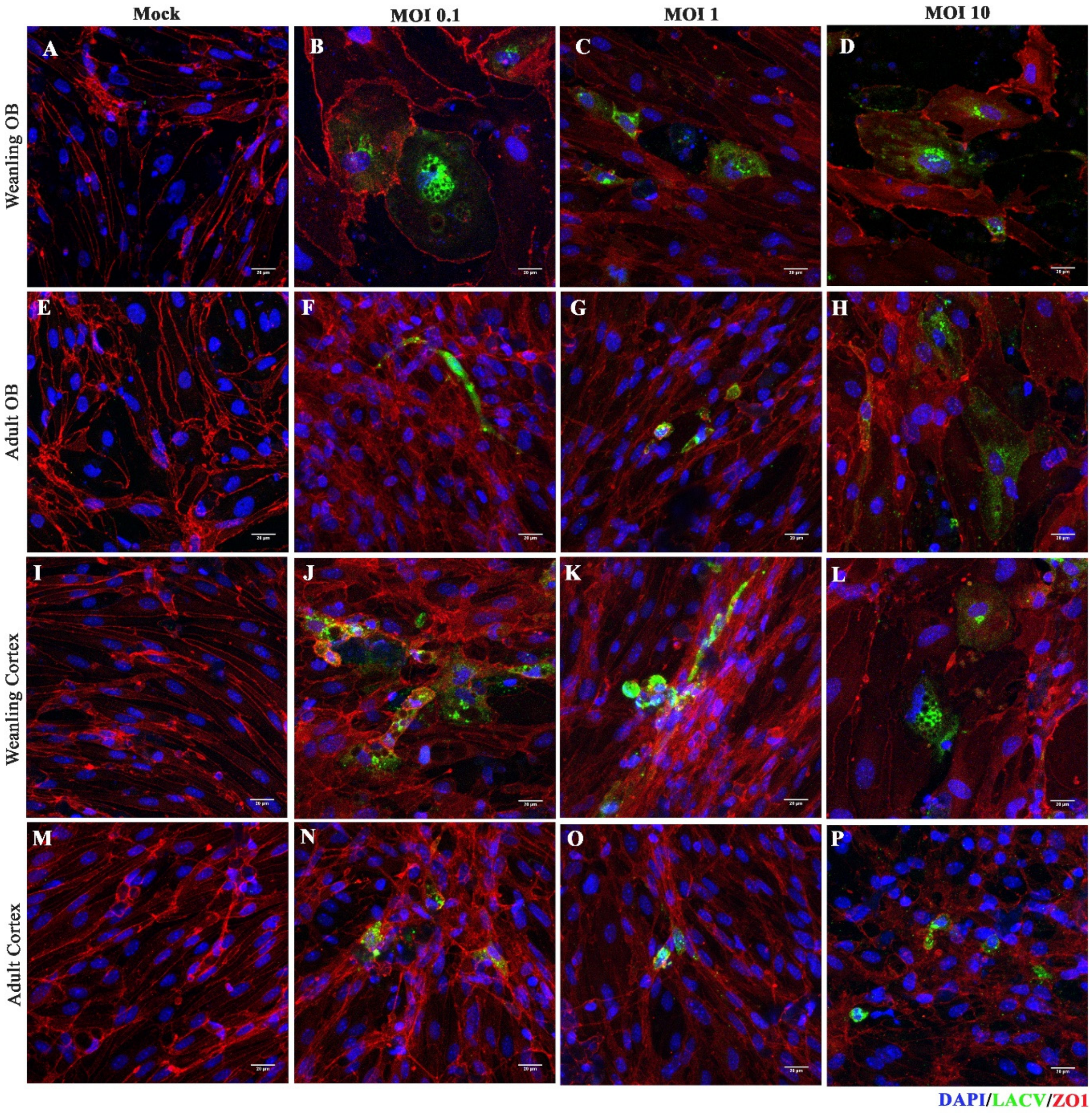

Mock
MOI 0.1
MOI 1
MOI 10
Weanling OB
Adult OB
Weanling Cortex
Adult Cortex
A
B
C
D
E
F
G
H
I
J
K
L
M
N
O
P
DAPI/LACV/ZO1

Supplementary fig. 4 Loss of weanling BCECs at 72 hpi after LACV infection observed at 40X objective magnification

Cultures described in supplementary image 3 were stained for DAPI (blue), LACV (green) and ZO1 (red) and imaged using 40X objective magnification . Weanling OB BCECs (A-D), adult OB BCECs (E-H) are shown. BCECs were mock-infected (A, E) or infected with LACV at MOIs of 0.1 (B, F), 1 (C, G) and 10 (D, H). Weanling cortical BCECs (I-L) and adult cortical BCECs (M-P) were mock-infected (I, M) or infected with LACV at MOIs of 0.1 (J, N), 1 (K, O), and 10 (L, P). Cells were stained with ZO1 (red), LACV (green) and nuclear counterstain DAPI (blue). Scale bar = 20 μm.

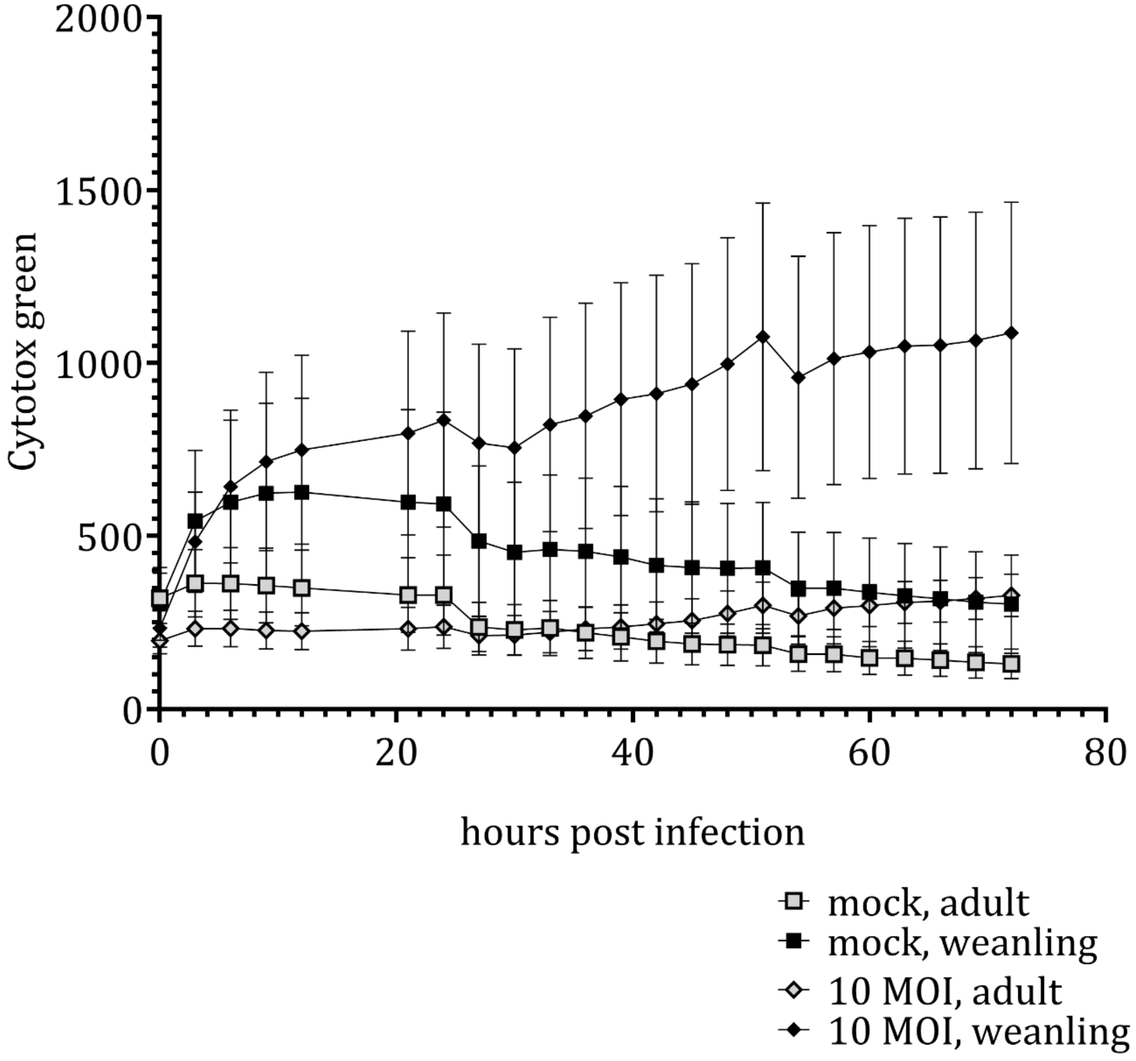

Cytotox green
2000
1500
1000
500
0
0
20
40
60
80
hours post infection
mock, adult
mock, weanling
10 MOI, adult
10 MOI, weanling

Supplementary fig. 5 Cell death assay and live cell imaging comparing weanling and adult BCECs infected with LACV

Weanling and adult BCECs were infected with LACV at 10 MOI or mock infected cells were maintained in parallel. After 1 h of infection, viral inoculum was removed, and Cytotox Green reagent was added, and the cells were imaged until 72 hpi using Incucyte live cell imager. The increment of green fluorescence (marker of cell death) is represented here. Data are shown as the mean green fluorescence per well for each 3 hrs. time for each group and standard error of the mean (N=3).

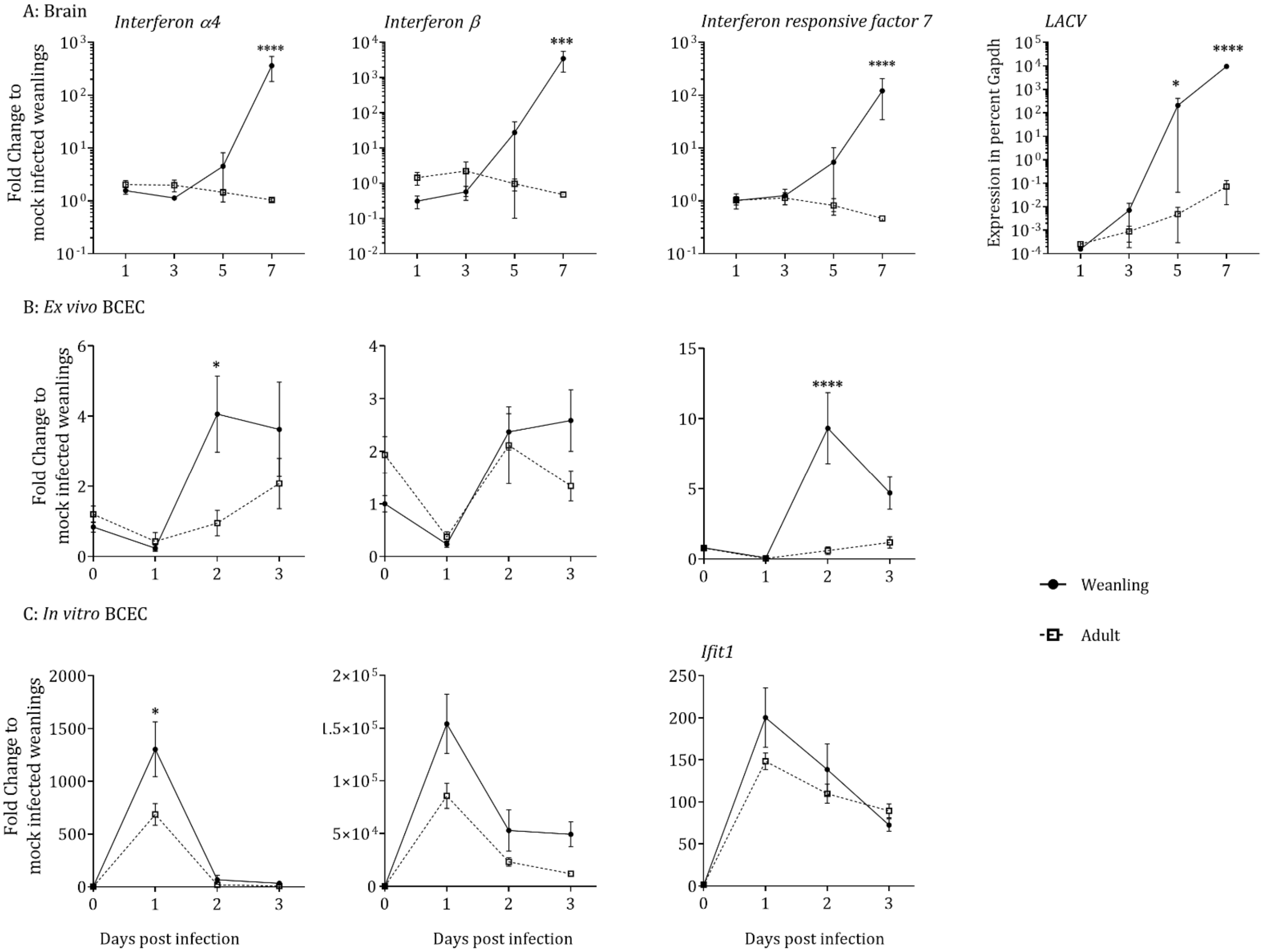

A: Brain
Interferon α4
Interferon β
Interferon responsive factor 7
LACV
Fold Change to mock infected weanlings
Expression in percent Gapdh
****
***
****
*
****
B: Ex vivo BCEC
*
****
C: In vitro BCEC
*
Ifit1
Days post infection
Weanling
Adult

Supplementary fig. 6 Innate immune gene response in brain or BCECs in LACV infection.

Adult and weanling mice were infected at 2000PFU/mouse and brains were obtained at 1, 3, 5- and 7-days post infection (dpi) (Panel A) and real-time PCRs were done for IFNs/ Interferon stimulated gene (ISG). *Ex vivo* extracted BCECs at 1, 2 and 3 dpi (Panel B) and *in vitro* inoculated BCECs at same dpi (Panel C) were also subjected to qRT PCR analysis for interferon α4 (*Ifna4*), interferon β (*Ifnb*), interferon responsive factor 7 (*Irf7*) or interferon-induced protein with tetratricopeptide repeats 1 (*Ifit1*) expression. For the brain samples LACV RNA was also analyzed. For the brain samples we used a log axis of expression and statistical analyses on that to demonstrate exponential induction of LACV RNA as well as other target genes, whereas *ex vivo* and *in vitro* cultured BCECs' expressions are shown and analyzed in linear axes.

Supplementary and video 1. Adult BCECs do not show widespread cell death.

Adult BCECs used in supplementary figure 5 were imaged using Incucyte live cell imager and a representative video of cell death is shown.

Supplementary and video 2. Weanling BCECs show higher amount of cell death than adult BCECs.

Weanling BCECs used in supplementary figure 5 were imaged using Incucyte live cell imager and a representative video of cell death is shown. Weanling BCECs have widespread cell death in LACV infection.
